# Brothers Building Brothers by Breaking Barriers: Protocol for a Pilot Trial of a Telehealth Social Capital Intervention for Young Black Sexual Minority Men Living With HIV

**DOI:** 10.2196/69961

**Published:** 2025-12-01

**Authors:** Marcus O Reed, McKinsey Bullock, Antonio Newman Jr, Srija Dutta, Samuel C O Opara, Tsedenia Tewodros, Kamini Doraivelu, Andrés Camacho-González, Raphiel Murden, Kristi Gamarel, Gary W Harper, Sophia A Hussen

**Affiliations:** 1 Hubert Department of Global Health Emory University Rollins School of Public Health Atlanta, GA United States; 2 Division of Infectious Diseases Department of Medicine Emory University School of Medicine Atlanta, GA United States; 3 Division of Infectious Diseases Department of Pediatrics Emory University School of Medicine Atlanta, GA United States; 4 Department of Biostatistics and Bioinformatics Emory University Rollins School of Public Health Atlanta, GA United States; 5 Department of Health Behavior and Health Equity University of Michigan School of Public Health Ann Arbor, MI United States

**Keywords:** social capital, resilience, HIV, intervention adaptation, YB-GBSMM, sexual minority health

## Abstract

**Background:**

Young Black sexual minority men are disproportionately affected by HIV, especially in the Southern United States. To address this, we developed Brothers Building Brothers by Breaking Barriers (B6) intervention with a goal of enhancing social capital and engagement in care among young Black sexual minority men living with HIV. However, we encountered challenges to feasibility in recruiting and engaging for an in-person intervention.

**Objective:**

The objectives of this study are to iteratively adapt the original B6 intervention for telehealth delivery (Phase 1), and pilot test the intervention through a waitlist-control trial to evaluate its feasibility, acceptability, and safety (Phase 2).

**Methods:**

In Phase 1, we used the assessment, decision, adaptation, production, topical experts, integration, training, and testing (ADAPT-ITT) framework to structure the iterative adaptation process of B6, working with a diverse study team and a Youth Advisory Board (YAB). After completing the preliminary adaptation process, we conducted initial Telehealth Brothers Building Brothers by Breaking Barriers (Tele-B6) pilot testing with a community partner organization. The result was a 5-week group-level intervention, delivered entirely remotely, consisting of a series of adapted activities to address bonding and bridging social capital, affirm intersectional identities, and engage in resilience-building processes. Following feedback integration from pilot-testing, we conducted Phase 2 with 60 young Black sexual minority men living with HIV recruited over the course of one year and who were randomized at the group level to either the immediate intervention or delayed (waitlist control) intervention group. Various data sources will be used to measure feasibility, acceptability, and safety, including surveys, postsession evaluation data, in-depth qualitative interviews, and review of medical records for HIV clinical outcomes.

**Results:**

Phase 1, the adaptation process of B6*,* began in fall 2022 and was completed in spring 2023. Phase 2, the implementation of the waitlist control trial, began in spring 2023 and concluded in summer 2024. Final follow-up assessments were completed in fall 2024 and the results of the mixed methods evaluation are expected in winter 2025.

**Conclusions:**

The adaptation process and telehealth delivery of B6 will add to the knowledge of strengths-based interventions designed to improve care engagement among young Black sexual minority men living with HIV.

**Trial Registration:**

ClinicalTrials.gov NCT05829759,https://clinicaltrials.gov/study/NCT05829759

**International Registered Report Identifier (IRRID):**

DERR1-10.2196/69961

## Introduction

### Background

In the United States, sexual minority men bear the highest burden of HIV. Within this population, Black sexual minority men are disproportionately impacted; in 2022, Black and African American males accounted for 77% of HIV cases among all Black and African American persons, with 80% being attributed to male-to-male sexual contact [1]. The severity of the HIV toll among Black sexual minority men is especially evident in the Southern United States, including Atlanta, Georgia; counties in the Atlanta area make up 4 of the 48 designated high-priority jurisdictions in the national Ending the HIV Epidemic initiative [2]. Young Black sexual minority men are additionally disproportionately affected by HIV; additional 2022 surveillance data indicate that individuals aged 25-34 years accounted for 40% of all new HIV cases, and 43% of new cases attributed to male-to-male sexual contact among Black males in the United States [1]. Moreover, this same report states that Black males aged 13-24 years accounted for 49% of cases among all male-to-male sexual contact [1].

Exacerbating these existing inequities, young Black sexual minority men are observed to experience lower rates of engagement along all stages of the HIV Continuum of Care (HIV-CoC) compared with other groups [3]. The HIV-CoC is a framework outlining the steps a person living with HIV follows and consists of the following stages: HIV testing and diagnosis, linkage to HIV care, retention in HIV care, antiretroviral medication adherence, and achievement and maintenance of viral suppression [4]. Young Black sexual minority men’s ability to successfully navigate the HIV-CoC is further complicated by broader, systemic factors at play. Structural racism and discrimination (including heterosexism and HIV stigma) create barriers to engagement across the HIV-CoC for young Black sexual minority men [5,6]. Specifically, HIV stigma within and outside of health care settings, logistical barriers such as insurance and transportation, and racism and discrimination that impact patient-provider relationships can all lead to decreased engagement of young Black sexual minority men across the HIV-CoC [7-10]. Social and environmental features of the Southern United States (eg, restricted Medicaid expansion, health care provider shortages, low health literacy, and higher community levels of stigma) can further impede young Black sexual minority men’s abilities to realize optimal health and lead fulfilling lives [11]. Despite the existing social and structural factors working against them, many young Black sexual minority men living with HIV overcome these existing barriers and are able to successfully engage in HIV care. Enhancing these resilience processes, or successful coping despite adversity, represents an important target for interventions to support young Black sexual minority men living with HIV.

### The Original (In Person) B6 Intervention Overview

Brothers Building Brothers by Breaking Barriers (B6) was created as an intervention to support resilience and ultimately improve engagement in HIV care for young Black sexual minority men living with HIV in Atlanta, Georgia [12]. The goal of B6 was to improve resilience processes by building social capital among participants [12]. The foundation of B6 was built to reflect an individual and network-level conceptualization of social capital, which is defined as the sum of benefits that can be derived from a person’s resource-containing, reciprocal, and trustworthy social network connections [13].

B6 was initially developed in 2017-2018 in Atlanta, Georgia, using principles of community-based participatory research, with input from young Black sexual minority men and community leaders throughout the intervention creation process [12]. However, we encountered significant challenges with recruitment and enrollment in our initial trial of B6 as an in-person intervention; many participants who agreed to join B6 did not ultimately attend the group intervention sessions despite multiple reminders and incentives [14]. We also received feedback that participants would prefer shorter sessions over a longer period. As such, the goal of this study is to adapt B6 to telehealth delivery using a videoconference platform (Zoom; Zoom Video Communications, Inc) to suit a post–COVID-19 environment, and to address transportation and other logistical barriers. This paper describes the adaptation and evaluation protocol for the new, telehealth version of B6 (Tele-B6).

### Theoretical Foundation

**Figure 1:**
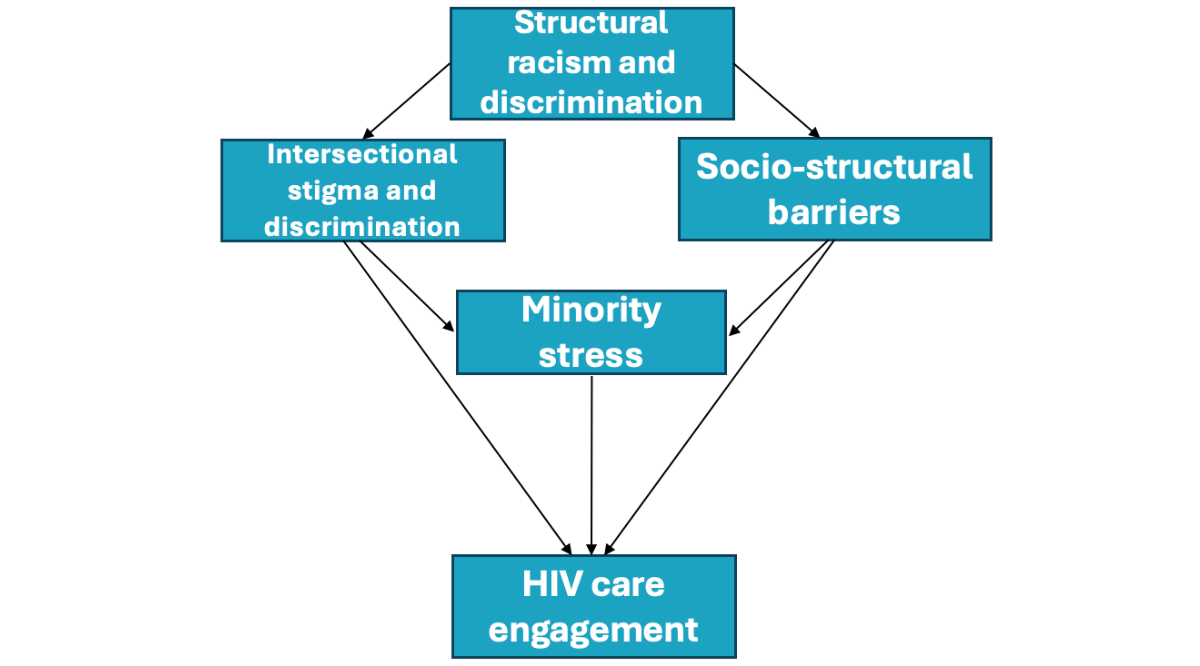
Study's conceptual model of theoretical relationships.

B6 is grounded in intersectionality, minority stress, social capital, and resilience concepts. Young Black sexual minority men living with HIV are in a unique societal position to simultaneously experience multiple intersecting layers of oppression and discrimination due to racism, heterosexism, and HIV stigma [15-17]. These external experiences with intersectional stigma and discrimination [18,19] decrease young Black sexual minority men’s access to power and are manifested in a range of socio-structural barriers (eg, poverty, incarceration, and exposure to violence) that fuel disparities across the HIV-CoC for young Black sexual minority men [20-22]. In addition to logistical and material challenges stemming from these intersecting social and structural barriers, additional adverse effects are experienced through psychological consequences of such discrimination, known as minority stress. The minority stress model posits that exposure to general stressors coupled with persistent stress stemming from prejudice, stigma, and discrimination places sexual minority individuals at increased risk for negative physical and mental health outcomes [23]. Minority stress can also be experienced intrapersonally through internalization of oppressive messaging, as illustrated by research documenting associations between internalized homonegativity, low self-esteem, and mental health comorbidities [24-26]. Psychological distress, including that resulting from minority stress and intersectional stigma, is in turn detrimental to HIV care engagement [9,27-30]. These foundational theoretical relationships are depicted in our study’s conceptual model (Figure 1).

Despite facing the multitude of barriers listed above, young Black sexual minority men living with HIV can and often do thrive [31,32]. Reorienting research to focus on this positive adaptation despite adversity [33,34], or resilience processes (a dynamic process in which individuals engage to positively adapt to adversity by using their assets and resources), represents a strength-based approach to address public health problems. The development of resilience plays an important role in buffering minority stress, including: (1) individual-level resilience processes, consisting of personality traits, and beliefs that help one cope with stress; and (2) community resilience processes, through which sexual minority communities provide social resources to facilitate coping amongst their members [35,36]. Many scholars have called for resilience-focused perspectives on understanding the health of sexual minority men [37-39]. However, although several studies describe resilience processes among young Black sexual minority men in the face of systemic oppression [32,40-45], few interventions specifically focus on building resilience processes as a strategy for enhancing HIV care engagement in this population [5]. Resilience is comprised of specific processes, which can serve as intervention targets: here, we focus on identity affirmation as an individual-level resilience process, and social capital as a community-level resilience process. As previously mentioned, these intervention targets also support our efforts to embrace a strengths-based approach, via undermining the influence of prime harmful manifestations of minority stress, such as practicing identity concealment and having poor social capital, both of which have been well demonstrated and observed amongst young Black sexual minority men.

### Identity Affirmation

Affirming beliefs about identity are important to individual-level resilience for racial and sexual minority youth. Positive beliefs about one’s racial identity (including the centrality of race to one’s self-concept, and the perception of how one’s race is viewed by others) are well-established contributors to resilience processes among Black youth [46,47]. The importance of affirming racial identity has been demonstrated among young Black sexual minority men, for whom positive racial identity beliefs have been shown to decrease risky sexual behavior [48]. In a qualitative study of young sexual minority men living with HIV (n=31/54, 57% Black), Harper et al [31] identified positive affirmation of racial and sexual orientation identity as key resilience processes. Moreover, 2 additional studies that also had a geographically diverse sample of youth, which were predominantly and all Black youth, respectively, revealed that youth who endorsed positive views of their racial identity reported fewer missed HIV care appointments; while those with higher levels of internalized homonegativity and HIV stigma missed more appointments [49,50]. Identity affirmation also facilitates access to community-level resilience resources; one must embrace their identity to connect with resources within a given minority community (eg, acknowledging one’s own gay identity or HIV status as a prerequisite to access lesbian, gay, bisexual, and transgender or HIV-focused organizations) [36].

### Social Capital

Social capital is a community-level resilience factor with potential to improve engagement across the HIV-CoC among young Black sexual minority men. Social capital can be defined as the sum of an individual’s resource-containing, reciprocal, and trustworthy social network connections [13,51] and is comprised of resources derived from social network connections. Social capital can improve HIV-CoC outcomes in several ways. For example, network contacts can educate one another about HIV, provide transportation assistance to appointments, and endorse health-promoting norms [52-56]. In a cross-sectional survey study, we previously demonstrated that social capital moderates the effect of depression on viral load suppression among young Black sexual minority men [57]. Others have similarly elucidated a buffering role of social capital in helping people cope with HIV stigma [53,58]. Poverty, trauma, mental health comorbidities, and structural racism can restrict young Black sexual minority men’s networks and social capital accumulation [58,59]. At the same time, unique community networks are well-described among young Black sexual minority men (eg, house and ball communities natural mentoring relationships), and can provide social capital to overcome logistical barriers to care [32,60-62].

### Study Objectives

The overall objective of this study is to adapt and pilot Tele-B6 as a strategy for enhancing feasibility and scalability before a larger efficacy trial. We seek to accomplish this objective via two specific aims: (1) to adapt B6 for telehealth delivery, in collaboration with young Black sexual minority men, community partners, and an advisory panel of subject matter experts; and (2) to conduct a pilot randomized controlled trial to evaluate feasibility, acceptability, and safety of the Tele-B6 intervention among young Black sexual minority men living with HIV in Atlanta.

## Methods

### Study Design

The current study will be completed in two phases. Phase 1, or the adaptation process of B6, has been completed. We used Wingood’s and DiClemente’s assessment, decision, adaptation, production, topical experts, integration, training, and testing (ADAPT-ITT) framework to inform and guide the adaptation process [63]. During this phase, the original B6 intervention was adapted for telehealth delivery, having incorporated feedback from the Youth Advisory Board (YAB) and other lessons learned from the original, in-person iteration of B6. Phase 2, the waitlist control pilot trial, has been completed. This study was conducted in line with SPIRIT (Standard Protocol Items: Recommendations for Interventional Trials) guidelines (checklist provided in Multimedia Appendix 1).

#### Phase 1: Adaptation Process for Telehealth Delivery

The ADAPT-ITT framework outlines a prescriptive method for adapting evidence-based interventions for new populations, including assessment, decision, adaptation, production, topical experts, integration, training, and testing. However, in the context of Tele-B6, the study setting and delivery method will change, while the target population remains unchanged (ie, young Black sexual minority men). We therefore used ADAPT-ITT as a more general guide to the adaptation of B6 into the current, telehealth version (Tele-B6), and focused our efforts on the later stages of the model (adaptation, production, topical experts, integration, training, and testing).

Adaptation and production: first, a team including research staff (2 young Black sexual minority men who cocreated the original B6 intervention and facilitated intervention sessions), the principal investigator, and a YAB member reviewed the manual closely and marked activities for deletion and consolidation or modification for the telehealth format. This subteam brainstormed potential modifications to each activity and drafted a new intervention manual for Tele-B6*.*

Topical experts: next, we convened the entire study team in person for a 2-day meeting, including additional study staff, investigators, and YAB members. We reviewed each activity and planned modifications in depth; thereafter, we tested new activities, and solicited feedback and ideas for any further modifications needed.

Integration, training, and testing: feedback from the 2-day full team meeting was integrated into a final manual. Supporting materials (eg, Microsoft PowerPoint presentations, worksheets, and websites) were developed and edited by the team. Next, we convened a group of Black sexual minority men through a community partner organization and conducted an initial pilot using videoconference. Feedback was solicited through a brief survey and additional modifications to procedures were made, resulting in the final Tele-B6 intervention*.* Finally, the 3 facilitators, all of whom identify as Black and gay persons, were prepared to lead weekly sessions by undergoing extensive training in group dynamics, which involved honing their active listening, conflict resolution, and interpersonal communication skills. Facilitators also familiarized themselves with the various features and functions of the Zoom platform to ensure they could navigate the platform as needed to conduct the intervention sessions and troubleshoot any technical issues that may arise.

#### Tele-B6 Intervention Description

Tele-B6 consists of weekly synchronous sessions, averaging 1-2 hours in length, in which young Black sexual minority men living with HIV engage in group discussions and activities via videoconference. Additional offline “homework” activities are also provided for participants to complete between sessions. Table 1 provides detailed information regarding Timing, Target constructs, Module title (in original B6), Module goals, and adaptations made for Tele-B6.

**Table 1:** Intervention content with adaptations from original in-person Brothers Building Brothers by Breaking Barriers to Telehealth Brothers Building Brothers by Breaking Barriers.

Timing and target constructs		Module title (in original B6^a^)	Module goals	Adaptations made for Tele-B6^b^
**Week 1: Introductions**				
		Set the stage	Introduce facilitators and participants, set expectations and create a safe space	Created a welcome video outlining the intervention’s ground rules and expectations; conduct an icebreaker via Zoom
	Social capital	Introduction to social capital	Explain the theoretical foundation of the intervention to participants	Updated original social capital presentation and supplemented with a humorous and engaging video to reinforce didactic material
	Identity affirmation	Who am I? Multiple identities	Identify the ways in which multiple identities (eg, gay identity, Black identity, HIV-positive identity) interact	Converted to telehealth group discussion; treat activity as “homework”^c^; created an example video to share with participants; and provided activity specific supplies in enrollment bag
**Week 2: Exploring self**				
	Social capital	Breaking communication barriers	Reflect on personal communication styles and any barriers to effective communication	Moved activity to Week 3; converted to telehealth group discussion
	Coping with minority stress	Critical self-reflection and coping skills	Reflect on how individual actions impact others; discuss common stressors and positive coping strategies	Converted to telehealth group discussion; divided participants into breakout rooms to afford robust discussion; and reinforce new skills
**Week 3: Building bonds**				
	Identity affirmation	Black gay slay: a history	Gain appreciation for the historical contributions of Black gay men in society	Moved this activity to Week 4; updated original “Black Gay Slay” presentation and B6 Jeopardy; emailed presentation to participants; treated activity (ie, presentation) as “homework”^c^
	Social capital	Black gay community	Explore perceptions, stereotypes and diversity within the Black gay community	Moved this activity to Week 4 and convert to telehealth discussion using a Zoom Whiteboard or Microsoft Word document
**Week 4: Building bridges part 1^d^**				
	Social capital	Navigating family	Gain tools to develop healthier relationships with family (birth or choice)	Converted to telehealth group discussion using a Zoom Whiteboard or Microsoft Word document
	Coping with racism and discrimination	Navigating professional spaces	Build skills for succeeding in professional arenas and cultivating relationships with colleagues and supervisors	Moved activity to Week 2; converted to telehealth group discussion
**Week 5: Building bridges part 2^d^**				
	Coping with racism and discrimination	Navigating clinical spaces	Enhance skills for navigating health care settings, share strategies for communicating effectively with providers	Converted to telehealth group discussion using a Zoom Whiteboard or Microsoft Word document
	Social capital	Navigating intimate relationships	Reflect on intimate relationships; learn strategies to improve partner communication.	Divided content between Weeks 2 and 3; converted to telehealth group discussion
**Week 5: Sustaining connections^e^**				
	Individual resilience	Individual goal setting	Set short and long-term goals for career, relationships, health.	Converted to telehealth group discussion using a Zoom Whiteboard or Microsoft Word document
	Social capital	Community action	Learn strategies for activism and formulate community action plans.	Converted to telehealth group discussion using a Zoom Whiteboard or Microsoft Word document
		Conclusions	Closing ceremony, distribution of a compilation of community resources	Distributed compilation of resources during enrollment visit; provided in participant manual

^a^B6: Brothers Building Brothers by Breaking Barriers.

^b^Tele-B6: Telehealth Brothers Building Brothers by Breaking Barriers.

^c^An activity treated as “homework” means that the activity was introduced to participants at the end of a session and later discussed the subsequent session.

^d^“Building bridges” material is covered in 2 weeks—weeks 4 and 5.

^e^“Sustaining connections” material is also covered in week 5.

#### Phase 2: Randomized Waitlist Control Trial

Phase 2 is a waitlist control trial of Tele-B6 with 60 young Black sexual minority men living with HIV. The waitlist control design was chosen because it offers an effective way to reconcile the need to have a control group while still ensuring all participants receive the intervention, to help maximize participants’ opportunities to reap potential study-associated benefits [64,65].

**Figure 2:**
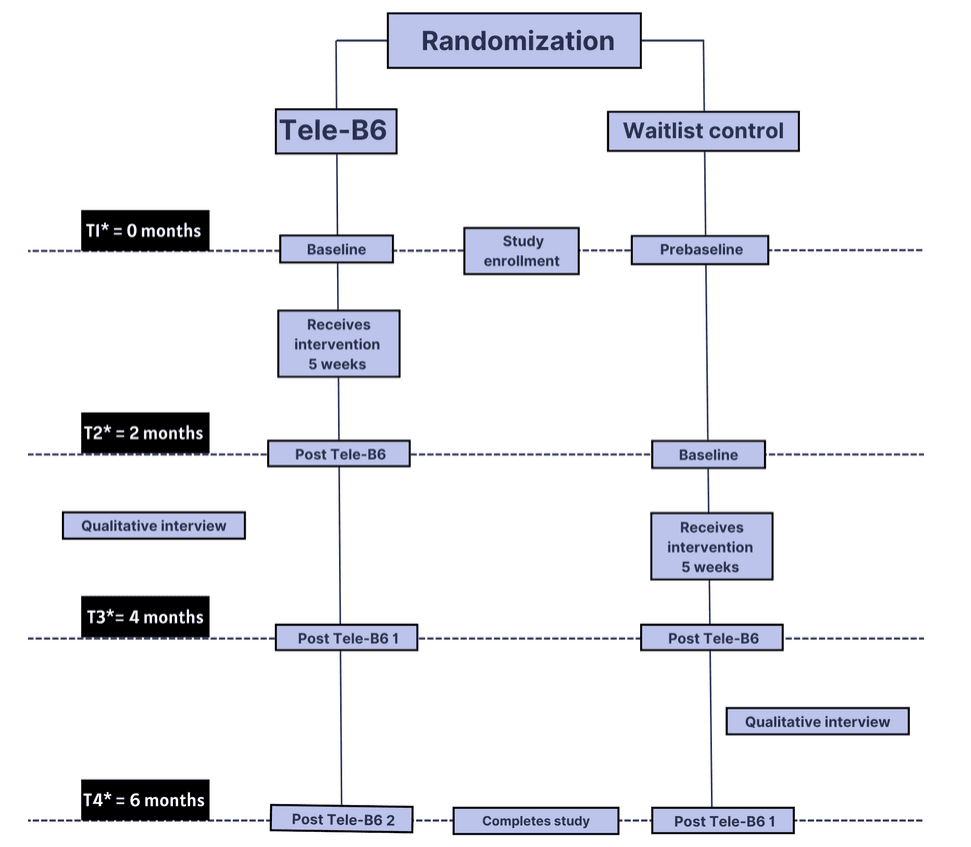
Telehealth Brothers Building Brothers by Breaking Barriers waitlist control study design schedule. Tele-B6: Telehealth Brothers Building Brothers by Breaking Barriers. T1-4: Time 1-4, at which participants are expected to complete the indicated study activities.

Following the enrollment visit, participants were randomized to either the immediate intervention (in which participants received the intervention within 2 weeks of their enrollment visit), or a delayed intervention (waitlist) condition, in which participants received the intervention 2 weeks after the 5-week program has concluded for the previous cohort of participants. See Figure 2 for the complete Tele-B6 waitlist control study design schedule.

### Participants

We recruited 60 participants. Participants completed an electronic screener survey housed in Research Electronic Data Capture (REDCap; Vanderbilt University) [66] to determine study eligibility. Eligible participants self-reported that they meet the following criteria: (1) Black race, inclusive of multiracial identities; (2) male gender identity, inclusive of transgender men; (3) self-identification as gay, bisexual, or nonheterosexual orientation, or any history of consensual anal or oral sex with men; (4) living with HIV; (5) age 18-29 years inclusive; (6) residence in the Atlanta Metropolitan Statistical Area; and (7) available and interested in meeting on the designated day and time over a 5-week period. Ineligible participants were excluded from study participation for the following reasons: (1) not living with HIV; (2) not having reliable internet access; (3) not satisfying the age requirement; and (4) not feeling comfortable participating in the study.

### Facilitators and Intervention Staff

The B6 sessions were facilitated by 3 full-time study staff members who identify as Black sexual minority men and thus share social identities with our participants. However, as sexual minority men are not a monolith, having 3 facilitators also allowed us to offer participants different perspectives and lived experiences to connect and identify with, which enhanced the facilitators’ rapport-building efforts with the participants. Of the 3 facilitators, one helped to develop and facilitate sessions for the in-person version of B6 (lead facilitator), and all 3 participated in the modifications of the intervention as well. In addition, the lead facilitator conducted trainings to ensure that all 3 were comfortable with procedures, intervention content, and evaluation procedures. Additional study team members joined the intervention sessions to provide technical assistance, and a physician member (who also identifies as a Black sexual minority men) of the study team was available for about half of the sessions to answer health-related questions that might arise, to improve the participants’ patient-provider communication skills.

### Setting

Enrollment and informed consent occurred in person at either the university or clinic research site*.* However, the intervention sessions for Tele-B6 were implemented via a secure teleconferencing platform (Zoom). The designated facilitators conducted the intervention from an office location on our university campus. The participants joined the intervention from any location of their choosing, and facilitators encouraged participants to join the intervention from a safe space that affords a reasonable level of comfort and privacy, given the sensitivity and nature of topics discussed (eg, experiences with discrimination, HIV serostatus, etc).

Participant recruitment has been completed. Most participants were recruited from a high-volume HIV care clinic located in Atlanta, Georgia. Other participants were recruited from advertisements on geosocial networking apps, from community-based partner organizations, and from venue-based recruitment at community locations frequented by young Black sexual minority men.

### B6 Program Materials

**Figure 3:**
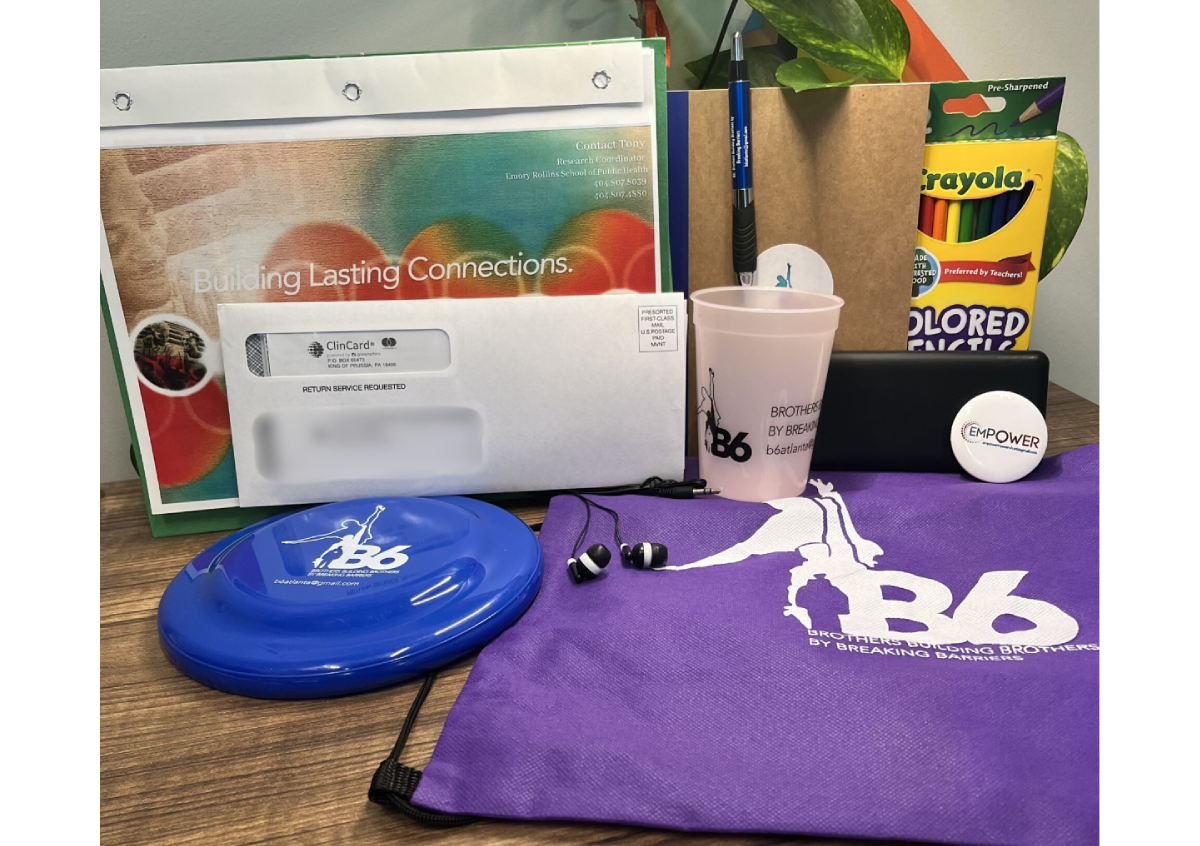
Telehealth Brothers Building Brothers by Breaking Barriers enrollment bag and program materials.

During the enrollment visit, participants received a drawstring backpack containing the following items (Figure 3):

1. A journal and pen with the study logo: to be used for “energizer” writing activities that took place at the beginning of certain synchronous sessions.
2. A pack of coloring pencils: this was used for the Mask activity (described below).
3. A pair of headphones: this was used to help afford participants a greater sense of privacy, if necessary.
4. A battery pack: this was used to help participants to keep their devices charged while participating in the program.
5. A participant manual: this folder contained a collection of handouts, worksheets, and activities necessary to complete the program, along with listings of resources and supports aimed to assist participants meet their immediate needs (eg, currently experiencing housing and food insecurity)
6. A ClinCard: participants were compensated for completion of activities, surveys, and weekly session attendance via a reloadable debit card.

In addition to the drawstring backpack, participants were granted access to a university-affiliated, auxiliary website (managed by the facilitators) to offer another point of access to the intervention materials. By making the intervention materials available electronically, we hoped this effort would at least minimize a barrier that could restrict participant engagement (ie, not having intervention materials readily available for the weekly synchronous sessions).

### Data Collection and Evaluation

We used a mixed methods approach to evaluate our intervention and assess our primary and exploratory outcomes**.** Data sources include an intervention log, adverse event reports, periodic electronic surveys, qualitative exit interviews, and EMR data. All data is housed in a secure location, with access to this data limited to our research team members. See Table 2 for a detailed outline of our mixed methods evaluation plan.

**Table 2:** Mixed methods evaluation plan.

Outcomes, constructs, and assessment strategy				Measures (where applicable)		Timing
**Primary outcomes**						
	**Acceptability**					
		Qualitative interviews	Open-ended questions adapted from original B6a exit interview guide		Exit interviews	
		Surveys	Postsession eval forms (adapted from original B6)		After each group session	
	**Feasibility**					
		Recruitment rates	Rates of participation from screened and eligible participants		Continuous	
		Retention	Attendance logs		Continuous	
		Intervention fidelity	Structured intervention log (adapted from original B6)		Continuous	
	**Safety**					
		Adverse events	Adverse event tracking forms		Continuous	
**Model constructs**						
	**Structural racism and discrimination**					
		Surveys	Measures of socioeconomic status, demographics Material resources scale [67]		0, 2, 4, 6 months	
	**Intersectional stigma and discrimination**					
		Surveys	Everyday Discrimination Scale [68] HIV Stigma Scale - 12 (HSS-12) [69]		0, 2, 4, 6 months	
		Qualitative interviews	Open-ended questions adapted from original B6 exit interview guide		Exit interviews	
	**Socio-structural barriers**					
		Surveys	Zip code to derive neighborhood measures		0, 2, 4, 6 months	
		Qualitative interviews	Open-ended questions adapted from original B6 exit interview guide		Exit interviews	
	**Individual resilience process: identity affirmation**					
		Surveys	Multidimensional Model of Black Identity [70] Lesbian, Gay and Bisexual Identity Scale [71] Identification and Involvement with Gay Community (IGCS) [72]		0, 2, 4, 6 months	
		Qualitative interviews	Open-ended questions adapted from original B6 exit interview guide		Exit interviews	
	**Community resilience process: social capital**					
		Surveys	Personal Social Capital Scale [51] Social Capital Investment Inventory (SCII) [73] Social Network Scale [74]		0, 2, 4, 6 months	
		Qualitative interviews	Open-ended questions adapted from original B6 exit interview guide		Exit interviews	
	**Minority stress**					
		Surveys	Mental health measures including: Depression (CESD-R) [75] General Well-Being (GWB) Generalized Anxiety Disorder-7 (GAD-7) [76] Post-Traumatic Stress Disorder (PTSD) Checklist [77]		0, 2, 4, 6 months	
		Qualitative interviews	Open-ended questions adapted from original B6 exit interview guide		Exit interviews	
**Clinical outcomes**						
	**HIV care engagement**					
		Electronic medical record (EMR) abstraction	HIV viral load Retention in Care (missed visits)		0 and 6 months	
		Surveys	Index of engagement in HIV care [78]		0, 2, 4, 6 months	

^a^B6: Brothers Building Brothers by Breaking Barriers.

### Primary Outcomes: Acceptability, Feasibility, and Safety

To assess acceptability, we created intervention satisfaction evaluation surveys and administered them at the end of each weekly group discussion. We also assessed acceptability using data on participants’ reactions to various program components gathered from the facilitators’ intervention log. Finally, we conducted exit interviews with selected participants at the end of the 5-week intervention period to gather qualitative feedback about the structure, content, and experience of the intervention. To assess feasibility, we monitored rates of outreach, recruitment, eligibility, enrollment, attendance, retention, and assessment completion. Our goals were to achieve intervention completion rates of >80% of group sessions completed, to maintain high intervention fidelity (goal>90% fidelity on checklists), and to ensure adequate retention across the follow-up surveys (goal>80%). Fidelity assessments were also an important part of feasibility assessments: Tele-B6 facilitators completed a structured intervention log after each session to assess fidelity to intervention, time needed, and feasibility of delivering the intervention curriculum as designed. To assess safety, we monitored adverse events, including psychological or physical events occurring over the course of intervention participation.

### Exploratory Outcomes

Although the primary aims of this study were to assess acceptability and feasibility, we also assessed HIV-CoC outcomes, as well as key mediators that are being targeted in Tele-B6. The selection of measures was guided by the relevant literature, our preliminary studies, and the conceptual model depicted in Figure 1.

Our eventual target outcome (in our subsequent, fully-powered study), HIV Care Engagement will be measured in terms of viral load suppression (defined as viral load measurement below 200 copies/mL at the most recent measurement before baseline, and at a data abstraction point at least 6 months post enrollment) and retention in HIV care, measured by missed visits before and after intervention participation.

Missed visits are a validated measure of retention [79]; additionally, we were not able to use the Department of Health and Human Services (DHHS) retention measure (>2 visits in 12 months, at least 3 months apart) [80] due to the limited follow-up period.

### Data Collection Procedures: Surveys

We used previously validated scales with excellent documented reliability among our study population to measure key constructs in our conceptual model (structural racism and discrimination, socio-structural barriers, intersectional stigma and discrimination, and minority stress) and the resilience processes (identity affirmation and social capital) targeted in Tele-B6*.* We used REDCap to create and administer baseline and follow-up surveys of participants. Surveys will be administered at baseline, 2-, 4-, 6-month timepoints as outlined above. Reminders to complete surveys were sent via text message and email where needed.

Survey Procedures: surveys were self-administered by the participants remotely. We estimated that the surveys take 30 minutes to complete. Upon completion of each survey, respondents received US $25 via a reloadable debit card (ie, ClinCard).

### Data Collection Procedures: Qualitative Interviews

To evaluate Tele-B6 feasibility and acceptability, as well as descriptive accounts of any impact on resilience processes and conceptual model constructs, we conducted qualitative exit interviews with a subset of participants (planned n=24 interviews). We conducted these in-depth interviews (IDIs) with a purposively selected sample of participants, including both participants who maintained high levels of engagement and those with lower levels of engagement. IDIs focused on experiences with the B6 intervention and recommended improvements. IDIs were conducted via Zoom by a trained study staff member who did not directly participate in the weekly sessions, in order to provide space for criticism if needed. Participants received an additional US $25 upon completion. All interviews were digitally recorded and uploaded to a secure server for professional transcription and analysis. The interviewer recorded field notes, documented salient themes to be discussed with the larger group, and explored in subsequent IDIs.

### Data Collection Procedures: Facilitator Evaluations and Intervention Log

Following each weekly session, facilitators evaluated the performances of fellow cofacilitators and themselves on standard postsession evaluation forms. This data were used to help facilitators identify improvement opportunities (eg, identifying technologically savvier approaches to deliver intervention material) and acknowledge their existing strengths (eg, having facilitators who have similar shared experiences as the participants helps build trust more easily among participants). All facilitator session evaluation data were logged onto a spreadsheet and housed with study-related data. In addition, an intervention log was maintained to track changes while still ensuring intervention fidelity.

### Data Collection Procedures: Clinical EMR Data Abstraction

To measure preliminary efficacy of Tele-B6 for improving engagement in care (defined using exploratory endpoints of care retention and viral suppression), we will abstract clinical data from the EMR. We will compare retention and viral suppression outcomes pre– and post–Tele-B6 and compare the intervention and control groups. At baseline and 6 months later, we will abstract viral load measurements as well as missed, scheduled, and attended appointments. Data will be entered into a secure REDCap database by trained abstractors (graduate research assistants), using standardized abstraction forms and manuals [81]. For the first 3 months of abstraction, EMR data will be abstracted by 2 study team members in parallel and examined for intercoder reliability. Differences will be discussed, and forms and manuals revised until intercoder reliability is adequate (κ> 0.80).

### Statistical Analysis

Our primary outcome is focused on descriptive analyses of the acceptability, feasibility, and safety indices. We will assess intervention target outcomes (ie, retention in care and viral suppression), hypothesized mediators (eg, social capital and identity affirmation), and background variables. These measures will be used to assess feasibility and acceptability (eg, acceptability of items and length of time to complete surveys) of data collection.

### Acceptability, Feasibility, and Safety

To evaluate the acceptability of the intervention, quantitative analyses of the intervention data will be descriptive and concentrate on tabulating and summarizing postsession satisfaction survey measures that feature both closed-ended and open-ended questions. As described below, we will also qualitatively analyze exit interviews conducted to assess acceptability and to identify any necessary modifications needed before a full trial. To evaluate the feasibility of the intervention, we monitored rates of recruitment and effort required (eg, number of staff hours), number of screenings conducted, and proportion eligible and agreed to enroll. We recorded the number of rescheduled, cancelled and missed sessions and assessment visits to inform estimation of staffing needs and retention protocols for a subsequent full trial. We will also assess the feasibility of verifying retention in care and viral load data by obtaining releases from participants to contact their provider or clinic by monitoring rates of verifiable EMR reports. To assess safety, we planned to tabulate and describe any reported adverse events that occurred during study participation; there were none.

### Qualitative Analysis

Exit interviews were digitally recorded, deidentified, and transcribed verbatim. To support rigorous and reproducible qualitative analysis, we will practice team coding and analysis using a pragmatic thematic analysis approach [82]. Analytic steps will include (1) after each interview, interviewers will document field notes including emerging topic areas for subsequent exploration; (2**)** transcripts will be entered into MAXQDA (VERBI Software GmbH) qualitative software for coding and organizing data [83]; (3) we will develop a preliminary codebook to include predetermined deductive codes related to theoretical domains of interest (eg, constructs from the conceptual model) and inductive codes that emerge from the data; (4) analysts will code a subset of transcripts and compare coding, with differences discussed in team meetings until consensus is reached; (5) similarities and differences across transcripts will be examined, and codes and themes revised accordingly. We will cease developing new codes when no new themes are seen; and (6) properties and dimensions of salient themes will be summarized and interpreted in detailed analytic memos (“thick descriptions”).

### Ethical Considerations

All Tele-B6 procedures discussed in this protocol were reviewed and approved by Emory University’s Institutional Review Board (IRB IRB00104876) and the Grady Research Oversight Committee. Eligible study participants provided informed consent to participate in this study and provided consent for study staff to access their electronic medical records (EMR) to corroborate data analyses. For security and confidentiality purposes, study data were deidentified and entered in REDCap, a Health Insurance Portability and Accountability Act (HIPAA) compliant data collection tool. During the enrollment visit, participants were informed that participation is voluntary and can withdraw from the study whenever requested. Also, throughout the duration of the study, participants were reminded of their ability to opt out of the study. Participant payments were deemed appropriate and fair by our local IRB. Participants were compensated via ClinCard (an automated and secure payment method for research participants); participants received US $50 for the enrollment visit, US $25 for each weekly synchronous session attended; US $25 for each follow-up survey; and US $25 for the optional qualitative interview.

## Results

**Figure 4:**
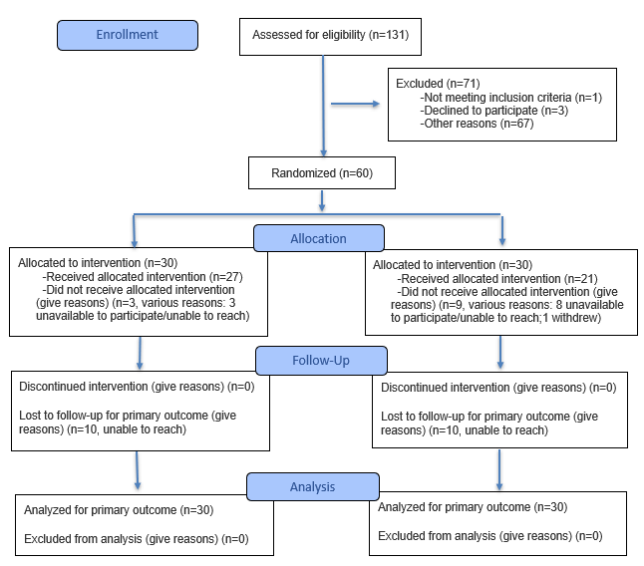
CONSORT (Consolidated Standards of Reporting Trials) flow diagram of study participants’ progress through each phase of a 2-arm randomized controlled trial.

Phase 1, the adaptation process of B6, began in fall 2022 and was completed in spring 2023. Phase 2, the implementation of the waitlist-controlled trial, began in spring 2023 and is still underway; delivery of Tele-B6 will conclude in summer 2024. Final results are expected in winter 2025 (Figure 4) [84].

## Discussion

### Principal Findings

Analyses of the feasibility, acceptability, and safety of the Tele-B6 program are currently underway. Our hypothesis is that Tele-B6 will demonstrate greater feasibility, and at least equivalent acceptability and safety, compared with the in-person version of the intervention. We further hypothesize that our procedures for measuring constructs of interest (eg, social capital and general well-being) will be acceptable to participants, and that our measures will be valid and reliable in this population. If these hypotheses are correct and the aims are achieved, we will seek to test the efficacy of the intervention through a larger, fully powered randomized controlled trial.

Tele-B6 addresses scientific gaps and answers calls from other scientists to develop resilience-based and culturally aware interventions for young Black sexual minority men. Boyd et al [85] found that external resilience assets (including family support, which is thematically related to social capital) were positively related to HIV-related health behaviors. Chang et al [86] found that young Black sexual minority men gained more from a health promotion intervention with community peers if discrimination was explicitly addressed, providing support for our culturally targeted, intersectional approach. Harper et al [87] similarly found promising preliminary results in decreasing sexual risk behavior in a pilot of a critical consciousness-based intervention, with explicit cultural and developmental tailoring, for young Black sexual minority men living with HIV. Tele-B6 has the potential to add to this small but growing toolbox of strategies designed specifically for and with young Black sexual minority men, as an effort towards ending the HIV epidemic.

There is a paucity of research dedicated to recognizing and addressing the social capital needs of young Black sexual minority men living with HIV. Such efforts are critically needed considering the pervasive barriers impeding HIV care engagement for young Black sexual minority men, and suboptimal engagement levels throughout the HIV-CoC. To this end, the adaptation process and implementation of Tele-B6 adds to the limited research of interventions centering resilience building and identity affirmation to enhance HIV-CoC engagement levels among young Black sexual minority men living with HIV in a post-COVID-19 context. Upon analyzing our data, we will plan to disseminate results to the larger scientific community (eg, through conference presentations and scientific manuscripts), as well as to our local community and study participants through in-person and webinar-based presentations.

### Conclusions

We have developed a remotely delivered version of a culturally tailored intervention, Tele-B6*,* which is designed to enhance the unique strengths of young Black sexual minority men who are living with HIV. Next steps will include mixed methods analysis to assess feasibility, acceptability, and safety. If our intervention meets these goals, we will seek to conduct a larger trial to test efficacy for improving mental health outcomes and engagement in medical care.

## Appendix

Multimedia Appendix 1 SPIRIT 2025 checklist.

Multimedia Appendix 2 Peer review report from the HIBI HIV/AIDS Intra- and Interpersonal Determinants and Behavioral Interventions Study Section, Risk, Prevention and Health Behavior Integrated Review Group (National Institutes of Health, USA).

## Abbreviations

ADAPT-ITT: assessment, decision, adaptation, production, topical experts, integration, training and testing
B6: Brothers Building Brothers by Breaking Barriers
DHHS: Department of Health and Human Services
EMR: electronic medical record
HIPAA: Health Insurance Portability and Accountability Act
HIV-CoC: HIV Continuum of Care
IDI: in-depth interview
REDCap: Research Electronic Data Capture
SPIRIT: Standard Protocol Items: Recommendations for Interventional Trials
YAB: Youth Advisory Board

## Data Availability

Data are available upon request. Data access is restricted to safeguard sensitive participant information.
