# Supplementary material for: Brothers Building Brothers by Breaking Barriers: Protocol for a Pilot Trial of a Telehealth Social Capital Intervention for Young Black Sexual Minority Men Living With HIV
Source: JMIR Res Protoc. 2025 Dec 1;14:e69961. doi: 10.2196/69961 (PMC12706449; doi:10.2196/69961)
Supplement: Multimedia Appendix 2 [file resprot_v14i1e69961_app2.pdf]

SUMMARY STATEMENT
(Privileged Communication)

PROGRAM CONTACT:
Susannah Allison
240-627-3861
allisonsu@mail.nih.gov

Release Date: 07/30/2021
Revised Date:

Application Number: 1 R34 MH129187-01

Principal Investigator

HUSSEN, SOPHIA A.

Applicant Organization: EMORY UNIVERSITY

Review Group: HIBI
HIV/AIDS Intra- and Inter-personal Determinants and Behavioral Interventions Study Section
AIDS - EXP. REV.

Meeting Date: 07/12/2021
Council: OCT 2021
Requested Start: 12/01/2021
RFA/PA: PA20-141
PCC: 9A-ASPA

Project Title: Brothers building brothers by breaking barriers (B6): A resilience-focused intervention for young Black gay and bisexual men living with HIV
SRG Action: Impact Score:21 Percentile:6 +
Next Steps: Visit https://grants.nih.gov/grants/next_steps.htm
Human Subjects: 30-Human subjects involved - Certified, no SRG concerns
Animal Subjects: 10-No live vertebrate animals involved for competing appl.
Gender: 3A-Only men, scientifically acceptable
Minority: 2A-Only minorities, scientifically acceptable
Age: 7A-Only Adults, scientifically acceptable

Project Year	Direct Costs Requested	Estimated Total Cost
1	150,000	239,325
2	150,000	239,325
3	150,000	239,325
TOTAL	450,000	717,975

ADMINISTRATIVE BUDGET NOTE: The budget shown is the requested budget and has not been adjusted to reflect any recommendations made by reviewers. If an award is planned, the costs will be calculated by Institute grants management staff based on the recommendations outlined below in the COMMITTEE BUDGET RECOMMENDATIONS section.

1R34MH129187-01 Hussen, Sophia

RESUME AND SUMMARY OF DISCUSSION: This application proposes to adapt an in-person intervention for young Black gay, bisexual and other men who have sex with men (YB-GBMSM) living with HIV, Brothers Building Brothers by Breaking Barriers (B6), that seeks to improve engagement in HIV care by focusing on intersectional identity affirmation and social capital. First, the team will use the ADAPT-ITT framework to adapt B6 so that it can be delivered as a telehealth intervention (tele-B6) within the setting of community-based organization (CBO). Next, a pilot RCT will evaluate the feasibility and acceptability of the tele-B6 intervention and also its impact on identity affirmation, social capital, and engagement in HIV care. Finally, the process of implementing the tele-B6 in the partner CBO will be evaluated. The target population of YB-GBMSM living with HIV is at high risk for HIV transmission and the proposed intervention could improve their engagement in HIV care. The study team is strong and the proposed intervention is based on the team's prior work with the B6 intervention. The committee noted only a few minor weaknesses that could be easily corrected and, therefore, the committee's overall enthusiasm for the potential impact of this application was very high.

DESCRIPTION (provided by applicant): Young Black gay, bisexual and other men who have sex with men (YB-GBMSM) encounter multilevel barriers to engaging in HIV care, as a direct result of structural and interpersonal racism, homonegativity and HIV stigma. These barriers lead to HIV disparities in which YB-GBMSM have high HIV prevalence and incidence, but suboptimal rates of engagement across the HIV Care Continuum. Resilience at both the individual and community levels can buffer the deleterious effects of stigma and discrimination on HIV care engagement. We have previously developed a culturally-specific resilience-building intervention for YB-GBMSM living with HIV called Brothers Building Brothers by Breaking Barriers (B6). B6 specifically targets intersectional identity affirmation (an individual-level resilience factor) and social capital (a community-level resilience factor) as a strategy for improving engagement in HIV care. The objective of this R34 application is to adapt B6, originally developed as an in-person intervention, for telehealth delivery within the context of a community-based organization (CBO). The rationale for the project is that telehealth delivery and CBO partnership will remove barriers to participation and facilitate scalability and sustainability. Our study will be based in Atlanta, Georgia – an HIV epicenter. This study will pursue three specific aims: (1) to adapt B6 for telehealth delivery, in collaboration with YB-GBMSM, community advisors, and subject matter experts; (2) to conduct a pilot randomized controlled trial (RCT) to evaluate feasibility and acceptability of tele-B6 among YB-GBMSM living with HIV in Atlanta; and (3) to evaluate the process of implementing B6 within the context of our partner CBO. For the first aim, we will use the ADAPT-ITT framework to convert B6 from an in-person to a telehealth intervention (tele-B6). For the second aim, we will test tele-B6 with N=60 YB-GBMSM and examine feasibility and acceptability of the intervention. We will also explore intervention impacts on social capital, identity affirmation, and HIV care engagement. In the third aim, we will utilize the Consolidated Framework for Implementation Research to examine the process of implementing tele-B6 within the structure of our partner CBO. The proposed research is highly significant because of its potential to develop a culturally relevant, scalable intervention for YB-GBMSM, a group who continues to be heavily and disproportionately impacted by HIV.

PUBLIC HEALTH RELEVANCE: The proposed project seeks to adapt and pilot test an intervention to build resilience among young Black gay, bisexual and other men who have sex with men (YB-GBMSM) who are living with HIV. This intervention is designed to improve young men's perceptions of their own identities, while also enhancing social connections and ultimately improving engagement in care. This project is relevant to public health because YB-GBMSM are disproportionately impacted by HIV, but face many barriers to care.

CRITIQUE 1

Significance: 1
Investigator(s): 1
Innovation: 3
Approach: 2
Environment: 1

Overall Impact: The impact of this proposed study on understanding the adaption of B6 for telehealth delivery within the context of a community-based organization (CBO) is likely to be strong in the field of HIV prevention science. HIV disproportionately impacts young Black gay, bisexual, and other men who have sex with men, particularly in the Southern region of the US. Structural racism and discrimination serve as key barriers to HIV care engagement for YB-GBMSM. YBGBMSM living with HIV are in a unique societal position to simultaneously experience multiple intersectional oppression and discrimination based on race, sexual identity, and HIV status. Resilience supports engagement across the HIV-CoC by buffering the detrimental impacts of structural racism, discrimination, and intersectional stigma. The scientific premise, telehealth delivery and CBO-based implementation of a resilience-informed intervention enhances social capital, affirms identity beliefs, and ultimately improves engagement across the HIV-CoC for YB-GBMSM, is well-conceptualized. The proposed study provides strong originality in the building on and extending the conceptualization of social capital, intersectionality, and resilience which serves as an innovative interdisciplinary framework for the adaptation of the B6 project. The investigative team demonstrates strong prior collaboration experience and expertise to conduct the proposed study. The delivery of a telehealth intervention through available videoconference technology and hosting resources in connection with a CBO website offers a novel, low-cost, low-tech strategy for scalability. It would be helpful to provide additional brief information about how intersectional identity experiences of YB-GBMSM relates to strategies for transcending barriers. It would be helpful to provide additional brief information about how social capital can be used as a strategy to improve HIV care engagement for YB-GBMSM. For the approach, the Youth Advisory Board, community partner, and Expert Advisory Panel reflect strong expertise and experience for the proposed study. The participatory processes with the Youth Advisory Board, community partner, and Expert Advisory Panel are well conceptualized. The adaptation process based on the ADAPT-ITT framework and intervention format/content are well conceptualized. The iterative CBPR modification process and facilitator training are clearly defined and operationalized based on the ADAPT-ITT framework. For Aim 2, the study design based on a wait-list control design for conducting a pilot randomized control trial is well presented. However, it would be helpful to provide additional brief detail about retention procedures for the proposed study, particularly since the target population experiences extensive structural barriers. The research team demonstrates excellent institutional resources to conduct the study.

1. Significance:

Strengths

- HIV disproportionately impacts young Black gay, bisexual, and other men who have sex with men, particularly in the Southern region of the US.
- Structural racism and discrimination serve as key barriers to HIV care engagement for YB-GBMSM. YBGBMSM living with HIV are in a unique societal position to simultaneously experience multiple intersectional oppression and discrimination based on race, sexual identity, and HIV status.

- Resilience supports engagement across the HIV-CoC by buffering the detrimental impacts of structural racism, discrimination, and intersectional stigma.
- The scientific premise, telehealth delivery and CBO-based implementation of a resilience-informed intervention enhances social capital, affirms identity beliefs, and ultimately improves engagement across the HIV-CoC for YB-GBMSM, is well-conceptualized.
- The proposed study provides strong originality in the building on and extending the conceptualization of social capital, intersectionality, and resilience which serves as an innovative interdisciplinary framework for the adaptation of the B6 project.

Weaknesses

- None noted.

2. Investigator(s):

Strengths

- The investigative team demonstrates strong prior collaboration experience.
- The investigative team reflects strong expertise to conduct the proposed study.

Weaknesses

- None noted.

3. Innovation:

Strengths

- The delivery of a telehealth intervention through available videoconference technology and hosting resources in connection with a CBO website offers a novel, low-cost, low-tech strategy for scalability.

Weaknesses

- It would be helpful to provide additional brief information about how intersectional identity experiences of YB-GBMSM relates to strategies for transcending barriers.
- It would be helpful to provide additional brief information about how social capital can be used as a strategy to improve HIV care engagement for YB-GBMSM.

4. Approach:

Strengths

- The implementation of the Youth Advisory Board is well-conceptualized.
- The Youth Advisory Board, community partner, and Expert Advisory Panel reflect strong expertise and experience for the proposed study.
- The participatory processes with the Youth Advisory Board, community partner, and Expert Advisory Panel are well conceptualized.
- The adaptation process based on the ADAPT-ITT framework and intervention format/content are well conceptualized.
- The iterative CBPR modification process and facilitator training are clearly defined and operationalized based on the ADAPT-ITT framework.

- For Aim 2, the study design based on a wait-list control design for conducting a pilot randomized control trial is well presented.

Weaknesses

- It would be helpful to provide additional brief detail about retention procedures for the proposed study, particularly since the target population experiences extensive structural barriers (e.g., poverty). For example, how will the CBO and Youth Advisory Board be utilized in strengthening retention.

5. Environment:

Strengths

- The research team demonstrates excellent institutional resources to conduct the study.

Weaknesses

- None noted.

Study Timeline:

Strengths

- The timeline is sufficient for the proposed research study.

Weaknesses

- None noted.

Protections for Human Subjects:

Acceptable Risks and/or Adequate Protections

Data and Safety Monitoring Plan (Applicable for Clinical Trials Only):

Acceptable

Inclusion Plans:

- Sex/Gender: Distribution justified scientifically
- Race/Ethnicity: Distribution justified scientifically
- For NIH-Defined Phase III trials, Plans for valid design and analysis: Not applicable
- Inclusion/Exclusion Based on Age: Distribution justified scientifically

Vertebrate Animals:

Not Applicable (No Vertebrate Animals)

Biohazards:

Not Applicable (No Biohazards)

Resource Sharing Plans:

Not Applicable (No Relevant Resources)

Budget and Period of Support:
Recommend as Requested

CRITIQUE 2

Significance: 2
Investigator(s): 2
Innovation: 2
Approach: 4
Environment: 2

Overall Impact: The proposed R34 aims to adapt the B6 intervention for telehealth delivery and will examine feasibility, acceptability and safety in 60 Young Black gay, bisexual and other men who have sex with men who are living with HIV in the Atlanta metro area. The study is highly significant in terms of its large population, setting and in its multilevel intervention approach. The team is an accomplished and established team - many of whom worked on the initial B6 intervention - who will be supported by an external advisory committee and will meaningfully include youth in the intervention adaptation. The approach is sound, with many of the decisions being justified by the team's previously collected data and experience in intervention development in youth. There are some, mostly minor, weaknesses in the approach including a lack of discussion about how the team with access EHR data, a generic description of the Aim 2 qualitative methods, and no *a priori* thresholds for the primary outcomes. However, these are weaknesses of omission and can be easily addressed. As such, the weaknesses only minimally reduce enthusiasm for this highly significant and innovative proposal.

1. Significance:
Strengths
- Young Black gay, bisexual and other men who have sex with men are a vulnerable and significant population at risk for HIV infection and low engagement in HIV care; that the study setting is in a EHE priority are further enhances the study's significance
- The thoughtful multilevel and strengths-based foci are likely to yield novel data that will have a positive impact on engagement in care in the team's population of interest
- The study premise is grounded in the team's previous study developing and pilot testing an in-person version of the B6 intervention; shift to a telehealth delivery mode is supported by their preliminary data
- If the study aims are accomplished, the resulting intervention could eventually (after further testing) advance the field of HIV engagement in care

Weaknesses
- None noted

2. Investigator(s):
Strengths

- Dr. Hussen is an established and productive investigator who has expertise in intervention development and HIV prevention among adolescents
- The extended team contains complementary expertise in mixed-methods research among Black adolescents (Harper) and community engaged HIV prevention research (Gamarel),
- The proposed team has previously collaborated on developing the CDC-funded B6 intervention (intervention to be adapted in the proposed study)

Weaknesses

- None noted

3. Innovation:

Strengths

- Embedding with the local CBO to help establish feasibility and efficacy is a novel and interesting approach to help speed up translation
- Other than the team's prior work, the focus on intersectional, multilevel resilience is an innovative intervention

Weaknesses

- No new methods or instrumentation are proposed

4. Approach:

Strengths

- Role of the CBO is well defined, will support the team in achieving the study aims, and is documented in their letter of support
- Study approach is wholly consistent with the principles of community-engaged research which, coupled with the team's previous use of these methods, will increase the study's likelihood of success
- Thoughtful and detailed description of how the team will use ADAPT-ITT
- Figure 2 is very helpful in illustrating the study design and participant flow
- Proposal identifies realistic limitations and includes strategies/discussion to mitigate them when possible

Weaknesses

- Unclear how engaging online synchronous group discussions are for the population, but this will be explored in Aim 1
- Minor but why will the team audio record the Zoom sessions when zoom has a record function?
- Eligibility criteria don't include being at a particular ID clinic so its not clear if/how the team will have access to medical records at all possible clinics in the Atlanta metro area. Similarly, the criteria don't include those who are not engaged in optimal HIV care/follow up; this may decrease the likelihood of the adapted intervention having an effect on the primary exploratory outcome
- There are no thresholds given for acceptability, feasibility or safety; instead, they will be tabulated and described

- Limited description of how the Aim 2 qualitative data will support next steps

5. Environment:

Strengths

- There are ample resources at Emory and the University of Michigan that should support the proposed project
- Letters of support indicate strong support for the proposed project by relevant partners

Weaknesses

- It is not described how the many resources listed at both institutions will directly support the proposed project

Study Timeline:

Strengths

- The three year timeline is consistent with the R34 mechanism and given the team's experience, and detailed approach, they are likely to complete the scope of work in that time
- Detailed timeline activities cover the span of work necessary to achieve the aims

Weaknesses

- None noted

Protections for Human Subjects:

Acceptable Risks and/or Adequate Protections

- Confidentiality of using a synchronous online platform, like zoom, is not discussed

Data and Safety Monitoring Plan (Applicable for Clinical Trials Only):

Acceptable

- No external safety monitor is proposed; it may be helpful to consider an external monitor in the case of AEs (though the risk is likely low)

Inclusion Plans:

- Sex/Gender: Distribution justified scientifically
- Race/Ethnicity: Distribution justified scientifically
- For NIH-Defined Phase III trials, Plans for valid design and analysis: Not applicable
- Inclusion/Exclusion Based on Age: Distribution justified scientifically
- Gender, race and age criteria are justified by the burden of the HIV epidemic in this population and the developmental tailoring of the B6 intervention

Vertebrate Animals:

Not Applicable (No Vertebrate Animals)

Biohazards:
Not Applicable (No Biohazards)

Resource Sharing Plans:
Acceptable

Budget and Period of Support:
Recommend as Requested

CRITIQUE 3

Significance: 1
Investigator(s): 1
Innovation: 1
Approach: 2
Environment: 1

Overall Impact: This R34 is to adapt and pilot a novel group-level intervention designed to affirm intersectional identities and augment social capital amongst young black gay, bisexual and other MSM (developed by the same team) for telehealth delivery. The proposed adaptation is responsive to results (including recognized limitations) of the prior pilot which was delivered in an in-person format. The team is well qualified to carry out the proposed work, and will build on existing community and institutional partnerships. Overall, this is a very well written proposal, plans are clearly articulated with details reflecting a deep understanding of the population, context as well as all aspects of research implementation.

1. Significance:

Strengths

- Focuses on young Black gay, bisexual and other MSM living with HIV in the US South – a population at very high risk of sub-optimal engagement in HIV care across the continuum
- The study will provide the requisite data for a planned R01 in which a hybrid implementation-effectiveness trial is planned
- The study builds on the team's prior work including the use of intervention content and assessments that have been used previously – the likelihood of success appears to be high

Weaknesses

- None noted by reviewer.

2. Investigator(s):

Strengths

- The study will be led by a strong team with the necessary / complementary expertise and experience with the population, intervention development, and trials implementation
- The team will be supported by a Youth Advisory Board & Expert Advisory panel

Weaknesses

- None noted by reviewer.

3. Innovation:

Strengths

- Focuses on building resilience as a strategy for enhancing HIV care engagement - a novel, strengths-based approach
- Focuses on intersectional identify experiences, directly addressing Black racial identify, sexual minority identity, and HIV status
- Use of telehealth format to deliver a resilience-focused intervention

Weaknesses

- None noted by reviewer.

4. Approach:

Strengths

- The overall study design is sound and appropriate for the R34 mechanism (adapt, test, explore process of implementation)
- Responsive to findings from prior work that showed the program to be highly acceptable with evidence of favorable effects on participants' self-reported stigma, internalized homonegativity, and engagement in HIV care – but also limitations due to challenges with recruitment and retention
- CBPR principles + ADAPT-ITT framework will be used to guide the adaptation process.
- The intervention will be pilot tested with a small sample (n=60), randomized to intervention or waitlist control groups
- Mixed-methods will be used to examine feasibility, acceptability and safety of the intervention
- Preliminary intervention effects will be explored – identity beliefs, social capital, stigma and HIV continuum of care engagement
- Proposes delivery via CBOs – this is a strength for multiple reasons (understanding of context, community, accessibility, and also potential scalability and sustainability). CFIR will be used to guide assessment.
- A multi-pronged recruitment approach will be used
- Twilio will be used to push brief messages / reminders
- Data collection will be done online using RedCap; best-practices for web-based data collection will be used

Weaknesses

- What are the thresholds for implementation outcomes?

5. Environment:

Strengths

- Strong environments to support the work

- Excellent local partnership (THRIVE SS)

Weaknesses

- None noted

Study Timeline:

Strengths

- Plans for recruitment and retention are sound
- Overall plan thoughtful and feasible

Weaknesses

- None noted by reviewer.

Protections for Human Subjects:

Acceptable Risks and/or Adequate Protections

Data and Safety Monitoring Plan (Applicable for Clinical Trials Only):

Acceptable

Inclusion Plans:

- Sex/Gender: Distribution justified scientifically
- Race/Ethnicity: Distribution justified scientifically
- For NIH-Defined Phase III trials, Plans for valid design and analysis: Not applicable
- Inclusion/Exclusion Based on Age: Distribution justified scientifically

Vertebrate Animals:

Not Applicable (No Vertebrate Animals)

Biohazards:

Not Applicable (No Biohazards)

Resource Sharing Plans:

Acceptable

Budget and Period of Support:

Recommend as Requested

THE FOLLOWING SECTIONS WERE PREPARED BY THE SCIENTIFIC REVIEW OFFICER TO SUMMARIZE THE OUTCOME OF DISCUSSIONS OF THE REVIEW COMMITTEE, OR REVIEWERS' WRITTEN CRITIQUES, ON THE FOLLOWING ISSUES:

PROTECTION OF HUMAN SUBJECTS: ACCEPTABLE

INCLUSION OF WOMEN PLAN: ACCEPTABLE

INCLUSION OF MINORITIES PLAN: ACCEPTABLE

INCLUSION ACROSS THE LIFESPAN: ACCEPTABLE

COMMITTEE BUDGET RECOMMENDATIONS: The budget was recommended as requested.

Footnotes for 1 R34 MH129187-01; PI Name: Hussen, Sophia A.

+ Derived from the range of percentile values calculated for the study section that reviewed this application.

NIH has modified its policy regarding the receipt of resubmissions (amended applications).See Guide Notice NOT-OD-18-197 at https://grants.nih.gov/grants/guide/notice-files/NOT-OD-18-197.html. The impact/priority score is calculated after discussion of an application by averaging the overall scores (1-9) given by all voting reviewers on the committee and multiplying by 10. The criterion scores are submitted prior to the meeting by the individual reviewers assigned to an application, and are not discussed specifically at the review meeting or calculated into the overall impact score. Some applications also receive a percentile ranking. For details on the review process, see http://grants.nih.gov/grants/peer_review_process.htm#scoring.

MEETING ROSTER

HIV/AIDS Intra- and Inter-personal Determinants and Behavioral Interventions Study Section
Risk, Prevention and Health Behavior Integrated Review Group
CENTER FOR SCIENTIFIC REVIEW
HIBI
07/12/2021 - 07/13/2021

Notice of NIH Policy to All Applicants: Meeting rosters are provided for information purposes only. Applicant investigators and institutional officials must not communicate directly with study section members about an application before or after the review. Failure to observe this policy will create a serious breach of integrity in the peer review process, and may lead to actions outlined in NOT-OD-14-073 at https://grants.nih.gov/grants/guide/notice-files/NOT-OD-14-073.html, NOT-OD-15-106 at https://grants.nih.gov/grants/guide/notice-files/NOT-OD-15-106.html, and NOT-OD-18-115 at https://grants.nih.gov/grants/guide/notice-files/NOT-OD-18-115.html, including removal of the application from immediate review.

CHAIRPERSON(S)

KIPKE, MICHELE D, PHD
PROFESSOR
DEPARTMENTS OF PEDIATRICS
AND PREVENTIVE MEDICINE
KECK SCHOOL OF MEDICINE
UNIVERSITY OF SOUTHERN CALIFORNIA
LOS ANGELES, CA 90028

MEMBERS

BUTLER, LISA MICHELLE, PHD
ASSOCIATE RESEARCH PROFESSOR
INSTITUTE FOR COLLABORATION ON HEALTH,
INTERVENTION, AND POLICY
UNIVERSITY OF CONNECTICUT
STORRS, CT 06269

COMULADA, WARREN SCOTT, DRPH
ASSOCIATE PROFESSOR
DEPARTMENT OF PSYCHIATRY
AND BIOBEHAVIORAL SCIENCES
SCHOOL OF PUBLIC HEALTH
UNIVERSITY OF CALIFORNIA, LOS ANGELES
LOS ANGELES, CA 90024

DODGE, BRIAN MARK, PHD
PROFESSOR
DEPARTMENT OF APPLIED HEALTH SCIENCE
INDIANA UNIVERSITY SCHOOL OF PUBLIC HEALTH
BLOOMINGTON, IN 47405

GRAHAM, SUSAN MARIE, MD, PHD
PROFESSOR
DIVISION OF ALLERGY AND INFECTIOUS DISEASES
DEPARTMENTS OF MEDICINE AND GLOBAL HEALTH
SCHOOL OF MEDICINE
UNIVERSITY OF WASHINGTON
SEATTLE, WA 98104

GROV, CHRISTIAN, PHD
PROFESSOR AND CHAIR
DEPARTMENT OF COMMUNITY HEALTH
AND SOCIAL SCIENCES
SCHOOL OF PUBLIC HEALTH AND HEALTH POLICY
CITY UNIVERSITY OF NEW YORK
NEW YORK, NY 10027

HANSEN, NATHAN B, PHD
DEPARTMENT HEAD AND PROFESSOR
DEPARTMENT OF HEALTH PROMOTION AND BEHAVIOR
COLLEGE OF PUBLIC HEALTH
UNIVERSITY OF GEORGIA
ATHENS, GA 30602

HORVATH, KEITH JOSEPH, PHD
ASSOCIATE PROFESSOR
DEPARTMENT OF CLINICAL PSYCHOLOGY
SAN DIEGO STATE UNIVERSITY
SAN DIEGO, CA 92120

IWELUNMOR, JULIET, PHD
ASSOCIATE PROFESSOR
DEPARTMENT OF BEHAVIORAL SCIENCE AND
HEALTH EDUCATION
COLLEGE FOR PUBLIC HEALTH AND SOCIAL JUSTICE
ST. LOUIS UNIVERSITY
ST. LOUIS, MO 63104

JOHNSON, DAWN M, PHD *
ASSOCIATE PROFESSOR
DEPARTMENT OF PSYCHOLOGY
UNIVERSITY OF AKRON
AKRON, OH 44325

LIPPMAN, SHERI ANN, PHD *
ASSOCIATE PROFESSOR
CENTER FOR AIDS PREVENTION STUDIES
UNIVERSITY OF CALIFORNIA, SAN FRANCISCO
SAN FRANCISCO, CA 94143

LOVEJOY, TRAVIS IAN, PHD
ASSOCIATE PROFESSOR
DEPARTMENT OF PSYCHIATRY
SCHOOL OF MEDICINE
OREGON HEALTH AND SCIENCE UNIVERSITY
PORTLAND, OR 97239

MACDONELL, KAREN KOLMODIN, PHD *
ASSOCIATE PROFESSOR
DEPARTMENT OF FAMILY MEDICINE
AND PUBLIC HEALTH SCIENCES
SCHOOL OF MEDICINE
WAYNE STATE UNIVERSITY
DETROIT, MI 48202

MUESSIG, KATHRYN E, PHD *
ASSISTANT PROFESSOR
DEPARTMENT OF HEALTH BEHAVIOR
GILLINGS SCHOOL OF GLOBAL PUBLIC HEALTH
UNIVERSITY OF NORTH CAROLINA AT CHAPEL HILL
CHAPEL HILL, NC 27599

OWCZARZAK, JILL, PHD *
ASSOCIATE PROFESSOR
DEPARTMENT OF HEALTH, BEHAVIOR AND SOCIETY
BLOOMBERG SCHOOL OF PUBLIC HEALTH
JOHNS HOPKINS UNIVERSITY
BALTIMORE, MD 21205

PATEL, VIRAJ V, MD, MPH *
ASSOCIATE PROFESSOR
DEPARTMENT OF MEDICINE
ALBERT EINSTEIN COLLEGE OF MEDICINE
BRONX, NY 10461

RAEL, CHRISTINE TAGLIAFERRI, PHD *
ASSISTANT PROFESSOR
COLLEGE OF NURSING
UNIVERSITY OF COLORADO ANSCHUTZ MEDICAL CAMPUS
AURORA, CO 80045

RAMSEY, SUSAN E, PHD
ASSOCIATE PROFESSOR
DIVISION OF GENERAL INTERNAL MEDICINE
RHODE ISLAND HOSPITAL
BROWN UNIVERSITY
PROVIDENCE, RI 02903

RODRIGUEZ-DIAZ, CARLOS EMANUEL, PHD *
ASSOCIATE PROFESSOR
DEPARTMENT OF PREVENTION AND COMMUNITY HEALTH
MILKEN INSTITUTE SCHOOL OF PUBLIC HEALTH
THE GEORGE WASHINGTON UNIVERSITY
WASHINGTON, DC 20052

SAFREN, STEVEN A, PHD
PROFESSOR
DEPARTMENT OF PSYCHOLOGY
COLLEGE OF ARTS AND SCIENCES
UNIVERSITY OF MIAMI
CORAL GABLES, FL 33124

SICONOLFI, DANIEL, MPH, PHD *
BEHAVIORAL SCIENTIST
RAND CORPORATION
PITTSBURGH, PA 15213

SSEWAMALA, FRED M, PHD
PROFESSOR
INSTITUTE FOR PUBLIC HEALTH
BROWN SCHOOL
WASHINGTON UNIVERSITY
ST. LOUIS, MO 63130

STOCKMAN, JAMILA KINSHASA, PHD
PROFESSOR
DIVISION OF GLOBAL PUBLIC HEALTH
DEPARTMENT OF MEDICINE
SCHOOL OF MEDICINE
UNIVERSITY OF CALIFORNIA, SAN DIEGO
LA JOLLA, CA 92093

SULLIVAN, PATRICK SEAN, PHD
PROFESSOR
DEPARTMENT OF EPIDEMIOLOGY
ROLLINS SCHOOL OF PUBLIC HEALTH
EMORY UNIVERSITY
ATLANTA, GA 30322

TANNER, AMANDA E, MPH, PHD *
ASSOCIATE PROFESSOR
DEPARTMENT OF PUBLIC HEALTH EDUCATION
SCHOOL OF HEALTH AND HUMAN SCIENCES
UNIVERSITY OF NORTH CAROLINA GREENSBORO
GREENSBORO, NC 27402

THAMES, APRIL D, PHD
ASSOCIATE PROFESSOR
DEPARTMENT OF PSYCHOLOGY
UNIVERSITY OF SOUTHERN CALIFORNIA
LOS ANGELES, CA 90089

THIELMAN, NATHAN M, MD, MPH *
PROFESSOR
DEPARTMENT OF MEDICINE
DUKE UNIVERSITY SCHOOL OF MEDICINE
DURHAM, NC 27710

WEBEL, ALLISON R, PHD
PROFESSOR
SCHOOL OF NURSING
UNIVERSITY OF WASHINGTON
SEATTLE, WA 98195

WILSON, TRACEY ELIZABETH, PHD *
PROFESSOR
DEPARTMENT OF COMMUNITY HEALTH SCIENCES
SCHOOL OF PUBLIC HEALTH
DOWNSTATE MEDICAL CENTER
THE STATE UNIVERSITY OF NEW YORK
BROOKLYN, NY 11203

WILTON, LEO, PHD, MPH
PROFESSOR
DEPARTMENT OF HUMAN DEVELOPMENT
COLLEGE OF COMMUNITY AND PUBLIC AFFAIRS
BINGHAMTON UNIVERSITY
BINGHAMTON, NY 13902

WINDSOR, LILIANE CAMBRAIA, PHD
ASSOCIATE PROFESSOR
SCHOOL OF SOCIAL WORK
THE UNIVERSITY OF ILLINOIS AT URBANA-CHAMPAIGN
URBANA, IL 61801

YBARRA, MICHELE L., PHD, MPH *
CEO AND RESEARCH DIRECTOR
CENTER FOR INNOVATIVE PUBLIC HEALTH RESEARCH
SAN CLEMENTE, CA 92672

SCIENTIFIC REVIEW OFFICER

RUBERT, MARK P, PHD
SCIENTIFIC REVIEW OFFICER
CENTER FOR SCIENTIFIC REVIEW
NATIONAL INSTITUTES OF HEALTH
BETHESDA, MD 20892

EXTRAMURAL SUPPORT ASSISTANT

CAMBRELEN, AMY ANGELA
EXTRAMURAL SUPPORT ASSISTANT
CENTER FOR SCIENTIFIC REVIEW
NATIONAL INSTITUTE OF HEALTH
BETHESDA, MD 20892

* Temporary Member. For grant applications, temporary members may participate in the entire meeting or may review only selected applications as needed.

Consultants are required to absent themselves from the room during the review of any application if their presence would constitute or appear to constitute a conflict of interest.
